# New Perspectives in Different Gene Expression Profiles for Early and Locally Advanced Non-Small Cell Lung Cancer Stem Cells

**DOI:** 10.3389/fonc.2021.613198

**Published:** 2021-03-31

**Authors:** Valentina Masciale, Federico Banchelli, Giulia Grisendi, Roberto D’Amico, Antonino Maiorana, Alessandro Stefani, Uliano Morandi, Massimo Dominici, Beatrice Aramini

**Affiliations:** ^1^ Division of Thoracic Surgery, Department of Medical and Surgical Sciences, University of Modena and Reggio Emilia, Modena, Italy; ^2^ Center of Statistic, Department of Medical and Surgical Sciences, University of Modena and Reggio Emilia, Modena, Italy; ^3^ Division of Oncology, Department of Medical and Surgical Sciences, University of Modena and Reggio Emilia, Modena, Italy; ^4^ Department of Medical and Surgical Sciences, Institute of Pathology, University of Modena and Reggio Emilia, Modena, Italy

**Keywords:** gene expression, target therapy, cancer stem cells, non-small cell lung cancer (NSCLC), early and advanced stage

## Abstract

**Introduction:**

Lung cancer is one of the most common cancers in the world, causing over 1.7 million deaths in 2018. Thus far, no effective treatments against lung cancer for advanced stages have been found. For early stages, although surgery is considered the gold standard treatment, 30–55% of patients develop recurrence within the first 5 years of surgery. Our aim is to assess whether cancer stem cells (CSC) display overexpression of a pool of genes that were previously identified for adenocarcinoma recurrence in patients with early and locally advanced stages of non-small cell lung cancer (NSCLC).

**Methods:**

This cross-sectional study was carried out by harvesting surgical tumor specimens obtained from patients harboring early (I-II) and locally advanced (IIIA) stages of NSCLC. For each patient, cell sorting was performed to identify and isolate the ALDH^high^ (CSC) and ALDH^low^ (cancer cells) populations. The mRNA expressions of 31 recurrence-related genes (target genes) in both ALDH^high^ and ALDH^low^ populations were then assessed and compared.

**Results:**

Surgical specimens were obtained from 22 patients harboring NSCLC. Sixteen (51.6%) out of 31 recurrence-related genes were significantly overexpressed in ALDH^high^ cells in the early stages and 9 (29.0%) were overexpressed in the locally advanced stages of NSCLC. Overall, the relative mRNA expressions for these recurrence-related genes were higher in early-stage patients. The average fold change, considering all 31 recurrence-related genes together, was 4.5 (95% CI = 3.1-6.3) in early-stage patients and 1.6 (95% CI = 1.2-2.2) in locally advanced-stage patients.

**Conclusions:**

Our study represents the first attempt toward identifying genes associated with recurrence that are overexpressed in cancer stem cells in patients with early and locally advanced stages of NSCLC. This finding may contribute to the identification of new target therapies tailored for NSCLC stages.

## Introduction

Lung cancer is one of the most common cancers in the world. In 2018, there were over 2 million new cases of lung cancer, and over 1.7 million deaths were attributed to lung cancer (1). In addition to traditional treatments, targeted therapy has emerged as an important means of disease management for patients with non-small cell lung cancer (NSCLC) (2). In particular, an improvement in NSCLC prognosis has been driven by immunotherapy alone or in combination with chemotherapy in both early and advanced stages, although a fraction of patients is still not responding with relevant issues in metastatic and, more recently, in early adjuvant settings (3–7). However, surgery is considered the gold standard for patients in the early-stages of lung cancer, even if 30–55% of patients develop recurrence within the first 5 years of surgery (1, 2, 8). The most updated guidelines in the eighth edition of the tumor, node, metastasis (TNM) staging system for lung cancer (9) defined early stages and operable locally advanced stages based on tumor dimensions and the number of lymph nodes and stations involved. Currently, no target treatment exists to prevent recurrence in early-stage cancer patients, and patients who have undergone lung resection have usually had a follow-up period of at least 5 years after surgery (8). Although adjuvant chemotherapy is often preferred in routine clinical practice, surgery may be delayed to a point that it is no longer feasible because of rapid tumor progression (10–12). On the other hand, neoadjuvant chemotherapy seems to offer several advantages, including, but not limited to, the ability to cure micrometastatic disease early, more effective drug delivery and tolerability, the ability to assess sensitivity to treatment, and the acquisition of prognostic information based on whether a major pathologic response has occurred (13–15). As a result, neoadjuvant chemotherapy should be regarded as an option for early-stage, operable NSCLC, as acknowledged by NCCN guidelines stating that “after surgical evaluation patients likely to receive adjuvant chemotherapy may be treated with induction chemotherapy as an alternative” (16, 17).

In recent years, medical oncologists have witnessed a revolution in the treatment of advanced NSCLC owing to the introduction of highly effective therapies, such as immunotherapy, for clinical use (18). Immunotherapy reverts the immune self-tolerance pathways through which a tumor avoids immune recognition and destruction. Currently, immune checkpoint inhibitors (ICIs) are widely used monoclonal antibodies in this field.

The success of immunotherapy in advanced NSCLC has prompted investigation of its use in earlier stages of the disease. In fact, possible challenges before or after surgery are under consideration in current clinical trials, although for the moment, the guidelines do not consider the introduction of immunotherapy as a neoadjuvant treatment for early stages (19, 20).

With regard to advanced stages, several clinical trials are attempting to establish new-generation target therapies (21, 22), although sufficient clarification has not been provided regarding which genes may be involved differently in early and advanced stages. We believe that this aspect is especially important and may represent a pillar for setting future lung cancer guidelines, including the discovery of molecular gene signatures (23) able to drive diagnostic and therapeutic approaches for both early and advanced stages. In addition, and just as importantly, recent scientific literature has focused on cancer stem cells (CSCs) (24), a class of pluripotent cells that has been observed in most types of solid and hematologic cancers involved in tumor growth, cell proliferation, and cancer cell dissemination for their self-renewal ability (25). This class of cells is also considered one of the main causes of resistance to standard medical treatments as chemotherapy and radiotherapy (24, 26), inducing “stemness” in cancer cells with a poor response to these therapeutic approaches (24, 26). Indeed, probably because of the difficulty of identifying and targeting CSCs, no study of lung cancer patients has tested the connection between these cells and recurrence. However, in recent decades, the scientific community has made a strong effort to identify the genes involved in recurrence of lung cancer cells (24). This research has been driven by scientists’ conviction that the ability to identify patients with a high rate of recurrence is crucial to reduce mortality from NSCLC (22, 27–29). The genes involved in lung cancer recurrence were identified several years ago in early genomic studies in NSCLC, showing an association between patient survival and gene expression profiles (23, 30–33). In addition, a signature of gene proliferation, derived from a meta-analysis on breast cancer data, was successfully translated to lung cancer (34). Park and colleagues demonstrated in 2012 that this prognostic signature has a strong component related to the cell cycle (35). In 2013, Wistuba et al. showed a prognostic signature of cell-cycle genes in adenocarcinoma of the lung. The cell-cycle proliferation (CCP) score was first applied to prostate adenocarcinoma, demonstrating its role as a strong predictor of death (36, 37). This score was tested retrospectively in three large independent cohorts of patients who had undergone surgery for lung cancer, testing 31 cell-cycle genes normalized by an average of 15 housekeeping genes. The combination of molecular and clinical data established that the CCP score has been shown as a reliable prognostic marker in the early stages of lung adenocarcinoma (28). Although the CCP signature is a superior prognostic tool in prostate cancer (36, 37), it is not surprising that many signatures lack robustness when applied to additional datasets. For that reason, other studies specifically in lung cancer patients have been carried out. In particular, in 2015, Raphael Bueno et al. (23), in collaboration with Myriad Genetics Inc., validated the CCP molecular expression signature in a large population. This confirmed that the signature could identify patients with a higher risk of cancer-related death after surgical resection of early-stage (I-II) lung adenocarcinoma (23). The researchers combined the CCP score and the pathological stage to predict deaths related to lung cancer. The genes selected and validated were those most involved in lung cancer relapse (23). This molecular prognostic score was then confirmed in a 2016 pilot study by Aramini B. and Myriad Genetics Inc. on 318 patients who underwent surgery for early-stage adenocarcinoma of the lung (29). The results confirmed the ability to stratify early-stage, resected lung cancer patients at risk of distant recurrence with the possibility of informing treatment and surveillance decisions (28, 29). However, this study focused on recurrence genes in human lung cancer cells. Considering the important role of CSC in recurrence (38–42), we decided for the first time to identify and extract cancer stem cells from human lung cancer patients who had undergone surgery for early or locally advanced NSCLC cancer, analyzing the same genes of recurrence previously studied in human lung cancer cells (23). The choice to use these genes derived from the fact that they have been previously validated in a larger cohort of patients (23).

In summary, the aim of this study was to assess whether cancer stem cells show overexpression of these recurrence-related genes, which were previously identified for adenocarcinoma recurrence (23), in patients with early and locally advanced stages of NSCLC.

We also believe that identifying genes that are expressed differently in early and advanced stages may be crucial to establish new target therapies and driving new diagnostic and treatment approaches; these approaches could be used both in early stages to better stratify patients and prevent recurrence and in locally advanced stages to employ targeted anti-CSC treatment for more effective cures.

## Methods

The study was conducted by harvesting surgical tumor specimens obtained from 22 patients harboring early (I-II) and locally advanced (IIIA) stages of NSCLC. For each patient, cell sorting was performed to identify and isolate the ALDH^high^ (CSC) and ALDH^low^ (cancer cells) populations. The mRNA expressions of 31 recurrence-related genes (target genes) in both ALDH^high^ and ALDH^low^ populations were then assessed and compared.

ALDH^high^ and ALDH^low^ quantification was measured as the percentage of viable cells according to cytofluorimetric analysis, and the expression of target genes was measured as a Ct value obtained by RT-PCR experiments.

### Study Design and Data

The present study was a cross-sectional study carried out in accordance with the guidelines for Strengthening the Reporting of Observational Studies in Epidemiology (STROBE) (43). The outcomes were the mRNA expressions of 31 target genes in ALDH^high^ and ALDH^low^ cell populations. The experimental RT-PCR data used for this research was deposited in the Gene Expression Omnibus public repository (https://www.ncbi.nlm.nih.gov/geo/) with access number GSE157427.

### Study Population

Patients who underwent major lung resection by lateral thoracotomy at the Division of Thoracic Surgery of Modena University Hospital (Italy) for stage I, II, or IIIA non-small cell lung cancer (tumor, node, metastasis (TNM) staging system, eighth edition) between October 2017 and September 2019 were included in the study. Inclusion criteria were ages between 18 and 85; R0 resection; availability of formalin-fixed, paraffin-embedded surgery specimens from the primary tumor; and availability of fresh surgical specimens for cytofluorimetric analysis. Exclusion criteria were incomplete resection; unknown tumor, node, and metastasis status; synchronous tumors; and previous lung cancer.

### Primary Cells Isolated From Human Lung Cancer

Surgical specimens were retrieved 1 to 2 h after surgery, washed in 50 mL sterile Falcon with Dulbecco’s phosphate-buffered saline (D-PBS) (L1825-BC—Merck Millipore, Italy), and mechanically minced into small pieces (2 mm to 4 mm). Samples were digested for 60 min at 37 °C in a gentle MACS Octo dissociator according to the manufacturer’s instructions, with Milteny tumor dissociation in a MACS™ C-Tube (Miltenyi Biotec, Italy). The cell suspension was then filtered through 70-μm sterile cell strainers, centrifuged at 300 × g for 5 min, and resuspended in a DMEM and HAM’S F12 media mixture (2:1) (Gibco) containing 50 IU/mL penicillin-streptomycin and 4 mM glutamine. Viable cells were counted using an optic phase-contrast microscope.

### Cell Sorting and Cytofluorimetric Analysis

The ALDH^high^ and ALDH^low^ cell populations were isolated through cell sorting. The protocol followed the instructions of the manufacturer (STEMCELL Technologies, Vancouver, BC). Primary tumor cell suspension from the surgical specimens was resuspended in ALDEFLUOR buffer containing BODIPY-aminoacetaldehyde at a concentration of one million cells/ml. Two tubes were labeled as “sample” and “control,” each containing 5 μl diethylamino-benzaldehyde (DEAB), which is a specific inhibitor of ALDH. Both control and test samples were incubated for 45 min at 37°C, protected from light. Following incubation, the cells were spun at 300 × g for 5 min. The cell pellet was resuspended in 1 ml ALDEFLUOR assay buffer. Following incubation, cell morphology was evaluated using side scatter (SSC) and forward scatter (FSC). Cell sorting and cytofluorimetric analysis were performed using a BD FACSAria III (Becton Dickinson, Franklin Lakes, NJ). The results were analyzed using fluorescence-activated cell sorting (FACS) Diva software (Becton Dickinson). The gating strategy included the ALDH^high^ gate, which was set at least one log apart from the ALDH^low^ gate. Sorted cells were promptly lysed for gene expression analysis.

### RNA Isolation and Retro Transcription

In accordance with the manufacturer’s instructions, RNA was extracted from both ALDH^high^ and ALDH^low^ cells using the RNeasy Mini Kit (Qiagen). Total RNA (500 ng) was reverse transcribed using the RevertAid™ First Strand cDNA Synthesis Kit (Thermo Scientific).

### Gene Expression Analysis

Gene expression analysis was performed on ALDH^high^ and ALDH^low^ cDNA samples using custom gene expression array on TaqMan low-density array (TLDA) cards. The cDNA was pre-amplified in duplicate. The pre-amplified cDNA samples and a control sample were diluted with Tris-EDTA (TE) buffer and combined with TaqMan Universal PCR Master Mix (ThermoFisher Scientific, Waltham, Massachusetts, USA). The resulting sample was loaded onto the array cards. The custom TaqMan™ gene expression assays (20X) (ThermoFisher Scientific) were pooled and diluted to 0.2X with Teknova TE (ThermoFisher Scientific). Gene expression was analyzed through different runs with one control for each run. Analysis of the pre-amplified cDNA was performed using ThermoFisher scientific custom TaqMan gene expression array cards (ThermoFisher Scientific) run on a QuantStudioTM 12K Flex Real-Time PCR system. Expression data were recorded as the cycle threshold (Ct) value, the PCR cycle at which the fluorescence intensity exceeds a predefined threshold.

The gene panel used for the analysis contained 31 target genes (36) (**Table 1**) and three housekeeping genes: RPL13A, RPL4, and RPS29. Of the 31 target genes, 20 (64.5%) were related to the cell cycle biological process, according to UniProt.

**Table 1:** List of target genes and associated biological processes.

Official symbol	Official Full name	Biological processes (UniProt)
ASF1B	Anti-Silencing Function 1B Histone Chaperone	Differentiation, Spermatogenesis, Transcription, Transcription regulation
ASPM	Assembly Factor For Spindle Microtubules	Cell cycle, Cell division, Mitosis
BIRC5	Baculoviral IAP Repeat Containing 5	Apoptosis, Cell cycle, Cell division, Chromosome partition, Mitosis, Transcription, Transcription regulation
BUB1B	Mitotic Checkpoint Serine/Threonine Kinase B	Apoptosis, Cell cycle, Cell division, Mitosis
C18orf24	Spindle And Kinetochore Associated Complex Subunit 1	Cell cycle, Cell division, Mitosis
CDC20	Cell Division Cycle 20	Cell cycle, Cell division, Differentiation, Mitosis, Neurogenesis, Ubiquitin-like conjugation pathway
CDCA3	Cell Division Cycle Associated 3	Cell cycle, Cell division, Mitosis, Ubiquitin-like conjugation pathway
CDCA8	cell division cycle associated 8	Cell cycle, Cell division, Mitosis
CDK1	Cyclin Dependent Kinase 1	Cell cycle, Cell division, DNA damage, DNA repair, Meiosis, Mitosis
CDKN3	Cyclin Dependent Kinase 3	Cell cycle
CENPF	Centromere Protein F	Cell cycle, Cell division, Differentiation, DNA synthesis, Mitosis, Myogenesis
CENPM	Centromere Protein M	Assembly of kinetochore proteins, mitotic progression and chromosome segregation
CEP55	Centrosomal Protein 55	Cell cycle, Cell division, Mitosis
DLGAP5	DLG Associated Protein 5	Cell cycle
DTL	Denticleless E3 Ubiquitin Protein Ligase Homolog	Biological rhythms, DNA damage, DNA replication, Ubiquitin-like conjugation pathway
FOXM1	Forkhead Box M1	Cell cycle, DNA damage, DNA repair, Transcription, Transcription regulation
KIAA0101	PCNA-associated factor	DNA damage, DNA repair
KIF11	Kinesin Family Member 11	Cell cycle, Cell division, Mitosis
KIF20A	Kinesin Family Member 20A	Protein transport, Transport
MCM10	Minichromosome Maintenance 10 Replication Initiation Factor	DNA damage, DNA replication
NUSAP1	Nucleolar And Spindle Associated Protein 1	Cell cycle, Cell division, Mitosis
ORC6L	Origin Recognition Complex, Subunit 6 Homolog-Like	DNA replication
PBK	PDZ Binding Kinase	Mitosis
PLK1	Polo-like Kinase 1	Cell cycle, Cell division, Mitosis
PRC1	Protein Regulator Of Cytokinesis 1	Cell cycle, Cell division
PTTG1	PTTG1 Regulator Of Sister Chromatid Separation	Cell cycle, Cell division, Chromosome partition, DNA damage, DNA repair, Mitosis
RAD51	RAD51 recombinase	DNA damage, DNA recombination, DNA repair
RAD54L	Recombination Protein RAD54-like	DNA damage, DNA repair
RRM2	Ribonucleotide Reductase Regulatory Subunit M2	DNA replication
TK1	Thymidine Kinase 1	DNA synthesis
TOP2A	DNA Topoisomerase II Alpha	Biological rhythms, Mitotic cell cycle G2/M transition decatenation checkpoint, Response to genotoxic stress, Up regulation of apoptosis, Activation of transcription from RNA polymerase II promoter

### GO Enrichment Analysis

GO analysis was used to identify characteristic biological processes (BP) for a large number of genes (39). The Reactome Pathway Browser (https://reactome.org/) and the Search Tool for the Retrieval of Interacting Genes (STRING, https://string-db.org/) were used to investigate enriched BP terms for all the genes that had differential relative mRNA expression in CSCs, comparing early and locally advanced NSCLC stages.

### PPI Network Construction and a Panel of Certified Gene Analysis

Protein-protein interactions (PPI) were assessed (44). These interactions can include direct (physical) and indirect (functional) associations. They arise from computational prediction, knowledge transfer between organisms, and interactions aggregated from other (primary) databases. Data were derived from five main sources: genomic context predictions, high-throughput laboratory experiments, conserved co-expression, automated text mining, and results of previous studies. STRING v. 11 was used to explore the interactive relationships of all the genes that had differential relative mRNA expression in CSCs, comparing early and locally advanced NSCLC stages. These relationships were reported as a PPI network. The number of edges in the network and the PPI enrichment *p*-value were calculated.

### Statistical Analysis

Continuous variables were described as mean ± standard deviation (SD) and range or as the median and interquartile range (IQR). Categorical variables were expressed as absolute and percentage frequencies. Correlations between target gene expressions were assessed by using Spearman’s rank correlation coefficient. A comparison of the observed correlations of gene expressions in ALDH^high^ or ALDH^low^ cell populations was conducted by using the Wilcoxon rank-sum test for all pairs of target genes.

Undetermined Ct values obtained from RT-PCR experiments were imputed. When one of the two duplicates had an undetermined Ct value, it was set as equal to the Ct of the other replicate. The remaining Ct values were then imputed as the maximum observed value for the specific target gene (45, 46). This was done only for patients who had at least one valid Ct value for that gene in ALDH^high^ or ALDH^low^ cells. Normalization of mRNA expression was performed by considering the expressions of three housekeeping genes (RPL13A, RPL4, and RPS29).

The relative mRNA expression of each of the 31 target genes was assessed, comparing ALDH^high^ to ALDH^low^ cell populations, by using linear mixed models (LMM) (47). The main analysis was carried out separately for patients with early-stage (I-II) NSCLC and locally advanced-stage (IIIA) NSCLC. Moreover, a secondary analysis was carried out by considering patients harboring adenocarcinoma or squamous cell carcinoma. The dependent variable in LMM was the Ct value, and the independent variables were ALDH (high vs. low), gene (target gene compared to the average of housekeeping genes), ALDH ^low^ gene interaction, histotype (adenocarcinoma or squamous cell carcinoma), and stage (early or locally advanced). The models also included a random intercept and a random ALDH^low^ gene interaction term that was specific for each patient, to account for correlations in individual Ct values. LMMs were estimated for each gene on those patients who had at least one available Ct value for that target gene in ALDH^high^ or ALDH^low^ cells. The results of LMMs were reported as fold changes (FC) and normalized differences in cycle thresholds (ΔΔCt equal to -log2 FC) with 95% confidence intervals (95% CI). As a summary measure, using the same statistical methodology, we also calculated the average relative mRNA expression considering all target genes together. This latter analysis aimed at measuring the unweighted difference between the average expression of the 31 target genes and the average expression of the 3 housekeeping genes; this calculation is similar to the calculation of the CCP score in (23).

A difference in relative mRNA expression was taken into account when the same target gene analyzed in both early stages and locally advanced stages showed an absolute value in ΔΔCt greater than 2. The same method was applied to compare target genes in both adenocarcinomas and squamous cell carcinomas.

To correct for multiple testing, the Benjamini-Hochberg false discovery rate (FDR) correction also was applied. Both raw and FDR-corrected *p*-values were reported for relative mRNA expression measures.

All statistical analyses were performed with R 3.4.3 software (The R Foundation for Statistical Computing, Wien) at a significance level of *p* < 0.05.

## Results

### Characteristics of Patients and Specimens

There were 22 patients with NSCLC who met the inclusion criteria (**Table 2**). Of them, 17 (77.3%) had adenocarcinoma, and 5 (22.7%) had squamous cell carcinoma. The average age was 70.0 ± 9.3 years (range = 52 - 84), 14 (63.6%) patients were males, and all were smokers or former smokers. Overall, clinical-stage IIIA (45.5%) was more frequent than stage I (31.2%) or stage II (22.7%). Furthermore, stage IIIA was observed in 8 (47.1%) adenocarcinomas and 1 (20%) squamous cell carcinoma.

**Table 2:** Characteristics of patients.

Characteristics of patients			
Age	Years	mean ± SD	70.0 ± 9.3
		median (IQR)	70 (63 - 75)
Gender	Male	n (%)	14 (63.6%)
Smoker^1^	Yes	n (%)	22 (100.0%)
Stage	I	n (%)	7 (31.2%)
	II	n (%)	5 (22.7%)
	IIIA	n (%)	10 (45.5%)
Histotype	Adenocarcinoma	n (%)	17 (77.3%)
	Squamous Cell Carcinoma	n (%)	5 (22.7%)
ALDH^high^ frequency	%	mean ± SD	4.0 ± 3.4
Tumor dimension	mm	mean ± SD	51.6 ± 23.4
Type of resection	Pneumonectomy	n (%)	3 (13.6%)
	Lobectomy	n (%)	19 (86.4%)
Surgical approach	Lateral Thoracotomy	n (%)	15 (68.8%)
	Video Assisted Thoracoscopy	n (%)	7 (31.2%)
Diagnostic procedure	Flexible Bronchoscopy	n (%)	11 (50.0%)
	Endobronchial Ultrasound	n (%)	11 (50.0%)

SD, standard deviation; IQR, interquartile range; ^1^including former smokers.

### Cancer Stem Cells Isolation

Cancer cells from lung surgical tumor specimens were efficiently isolated, as proven by the FSC and SSC values during morphology evaluation at cytofluorimetric analysis. The viability of the samples was good based on 7-AAD staining. The CSCs were physically isolated by FACS from the bulk parental tumor cell population and recovered according to the gating strategy described by Masciale et al. (48). An ALDH^high^ subpopulation was identified in all samples; on average, this subpopulation was 4.0% ± 3.4% (range 0.4%–12.5%) of all viable lung cancer cells.

### Descriptive Analysis of RT-PCR Data

There were 980 valid Ct values for target genes from the RT-PCR analyses. An additional 192 undetermined Ct values were imputed as being equal to their duplicate values, and 252 were imputed as the maximum observed Ct value for the specific gene.

**Figure 1:**
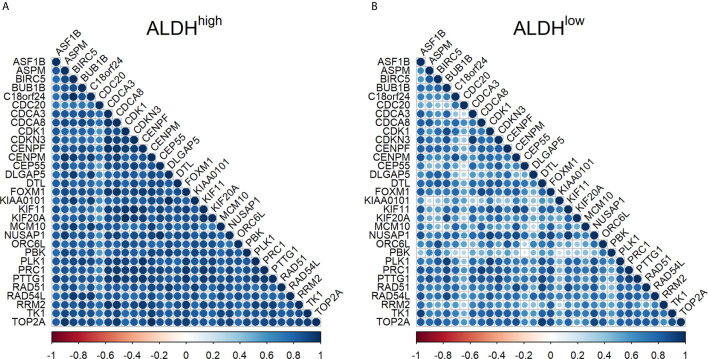
Correlation between mRNA expression of target genes in ALDH^high^ and ALDH^low^ cells in non-small cell lung cancer. **(A)** correlation in ALDH^high^ cells; **(B)** correlation in ALDH^low^ cells. The Spearman’s correlation coefficient for Ct values is reported for each pair of target genes by using a circle. The color and the diameter of the circles are proportional to the intensity of the correlations, as reported in the color palette. Blue indicates a highly positive correlation, white indicates no correlation, and red indicates a highly negative correlation.

A high correlation was found among the Ct values of the target genes in ALDH^high^ cells, with a median Spearman’s correlation coefficient equal to 0.86 (IQR = 0.81-0.90). In ALDH^low^ cells, the median Spearman’s correlation coefficient was 0.72 (IQR = 0.58-0.80). This was significantly lower than that of ALDH^high^ cells (*p <.*0001 by Wilcoxon test) (**Figure 1**). The genes that were less correlated to the other genes in ALDH^low^ cells were CDC20, KIAA0101, and PBK.

### Relative mRNA Expression of Target Genes in Early and Locally Advanced Stages of NSCLC

**Figure 2:**
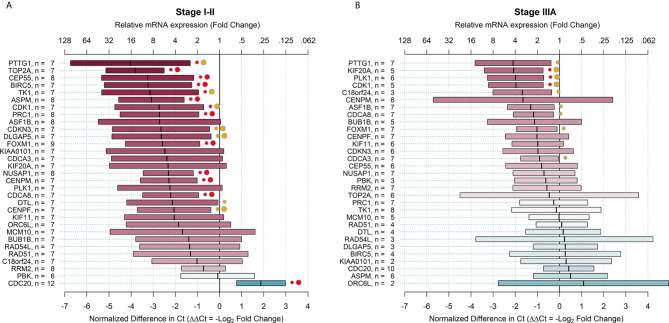
Relative mRNA expression of target genes in ALDH^high^ cells compared to ALDH^low^ cells in early and advanced stages of non-small cell lung cancer. The plots show relative mRNA expression measures of 31 recurrence genes, comparing ALDH^high^ to ALDH^low^ cells populations. The red color of boxes indicates a higher expression in ALDH^high^ cells, whereas the blue color indicates a higher expression in ALDH^low^ cells. **(A)** early NSCLC stages (I-II); **(B)** advanced NSCLC stages (IIIA). Ct = cycle thresholds. ΔΔCt = Delta Delta Ct (normalized difference in Ct). The mid-points of the boxes are the punctual estimates and the boxes represent the 95% confidence intervals (not adjusted for multiple comparisons). The level of statistical significance is reported as red (p < 0.01) or orange (p < 0.05) circles next to the boxes. Small circles represent raw p-values, whereas large circles represent Benjamini-Hochberg-corrected p-values (i.e. with false discovery rate (FDR) correction). There were 16 (51.6%) target genes that were significantly overexpressed in the early stages and 9 (29.0%) genes significantly overexpressed in the advanced stages of NSCLC. When the FDR correction was applied for multiple comparisons, the number of overexpressed genes declined to 15 (48.4%) in early stages and 3 (9.7%) in advanced stages.

The relative mRNA expression, comparing ALDH^high^ to ALDH^low^ cell populations in early and locally advanced stages of NSCLC, is shown in **Figure 2**. The results showed that 28 (90.3%) genes in the early stages of NSCLC and 11 (35.5%) genes in locally advanced stages of NSCLC were overexpressed in ALDH^high^ cells with FC > 2, regardless of statistical significance. Furthermore, 16 (51.6%) target genes were significantly overexpressed in ALDH^high^ cells in the early stages (PTTG1, TOP2A, CEP55, BIRC5, TK1, ASPM, CDK1, PRC1, CDKN3, DLGAP5, FOXM1, NUSAP1, CENPM, CDCA8, DTL, and CENPF), and 9 (29.0%) genes in locally advanced stages of NSCLC were overexpressed (PTTG1, KIF20A, PLK1, CDK1, C18orf24, ASF1B, CDCA8, FOXM1, and CDCA3). Overall, the relative mRNA expressions for these latter genes were higher in early-stage patients. Indeed, the FC values ranged from 16.3 for PTTG1 to 4.2 for CENPF in early-stage patients and from 4.3 for PTTG1 to 1.9 for CDCA3 in locally advanced-stage patients (**Figure 2**). The average FC, considering all 31 target genes together, was 4.5 (95% CI = 3.1-6.3, *p*-value <.0001) in early-stage patients and 1.6 (95% CI = 1.2-2.2, *p*-value = .0010) in locally advanced-stage patients; the difference between them was statistically significant (*p*-value of the interaction term <.0001). When the FDR correction was applied for multiple comparisons, the number of overexpressed genes declined to 15 (48.4%) in the early stages and 3 (9.7%) in locally advanced stages. To describe the internal consistency within the early-stage subgroup, we also compared the ΔΔCt values between stage I and stage II patients. The average ΔΔCt in stage I patients was -1.60 (95% CI = -1.02; -2.17, *p*-value <.0001) and -2.64 (95% CI = -1.90; -3.38, *p*-value <.0001) in stage II patients; their difference was slightly not statistically significant (*p*-value of the interaction term = 0.0550). Finally, there were 10 target genes that had a differential relative mRNA expression (difference in ΔΔCt > 2) comparing patients with early and locally advanced stages of NSCLC were compared (ASPM, BIRC5, TOP2A, TK1, ORC6L, DLGAP5, KIAA0101, PRC1, CEP55, and DTL) (**Figure 2**). All of these genes showed higher relative mRNA expression in early-stage NSCLC.

### Relative mRNA Expression of Target Genes in Adenocarcinoma and Squamous Cell Carcinoma

**Figure 3:**
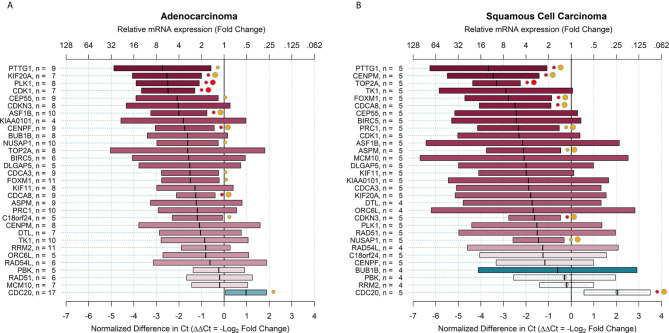
Relative mRNA expression of target genes in ALDH^high^ cells compared to ALDH^low^ cells in all stages of adenocarcinoma and squamous cell carcinoma. The plots show relative mRNA expression measures of 31 recurrence genes, comparing ALDH^high^ to ALDH^low^ cells populations. The red color of boxes indicates a higher expression in ALDH^high^ cells, whereas the blue color indicates a higher expression in ALDH^low^ cells. **(A)** all stages adenocarcinoma; **(B)** all stages squamous cell carcinoma. Ct = cycle thresholds. ΔΔCt = Delta Delta Ct (normalized difference in Ct). The mid-points of the boxes are the punctual estimates and the boxes represent the 95% confidence intervals (not adjusted for multiple comparisons). The level of statistical significance is reported as red (p < 0.01) or orange (p < 0.05) circles next to the boxes. Small circles represent raw p-values, whereas large circles represent Benjamini-Hochberg-corrected p-values (i.e. with false discovery rate (FDR) correction). There were 12 (38.7%) target genes that were significantly overexpressed in adenocarcinoma and 6 (19.4%) genes overexpressed in squamous cell carcinoma. When the FDR correction was applied for multiple comparisons, the number of overexpressed genes declined to 9 (29.0%) in adenocarcinoma and 9 (29.0%) in squamous cell carcinoma.

The relative mRNA expression in all stages of adenocarcinoma and squamous cell carcinoma is shown in **Figure 3**. On average, the relative expression measures did not differ greatly between the two subgroups, in which only two target genes had ΔΔCt that differed by more than 2 Ct (CENPM and TK1). The estimates for squamous cell carcinoma were less precise because of the smaller sample size.

### GO Enrichment Analysis

**Figure 4:**
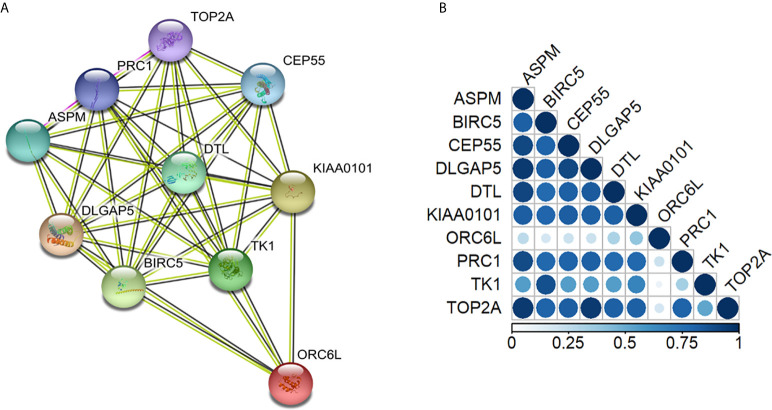
Protein annotations of genes with differential relative mRNA expression in early- and advanced-stage non-small cell lung cancer stem cells. **(A)** STRING diagram representing a protein interaction pathway derived from 10 genes identified as overexpressed in lung CSCs (https://string-db.org/). The network was made by 41 edges with a protein-protein interaction enrichment p-value < 0.0001. **(B)** Gene co-expression analysis revealed that in Homo Sapiens, all the genes were highly co-expressed except for the ORC6L gene. In the triangle matrix, the color indicates the level of co-expression of pairs of proteins in Homo sapiens. The co-regulation map can be explored at www.proteomeHD.net.

All 10 proteins derived from the 10 differentially overexpressed genes in CSCs were analyzed, and early and locally advanced NSCLC stages were compared. The PPI network contained 10 nodes and 41 edges with a PPI enrichment *p*-value <.0001. This indicated that these proteins were involved in the same biological processes (**Figure 4**), except for ORC6L, which was not as closely related to the other proteins.

When patients with early and locally advanced stages of NSCLC (**Table 3**) were compared with regard to these 10 proteins differentially overexpressed in CSCs, all of these proteins were found to be involved in the cell cycle and cell cycle regulation (**Table 3**).

**Table 3:** GO enrichment analysis.

GO:Term	Description	Count in gene set	p-value^1^
GO:0022402	cell cycle process	8 of 890	0.0000
GO:1903047	mitotic cell cycle process	6 of 564	0.0000
GO:0000280	nuclear division	5 of 268	0.0000
GO:0051726	regulation of cell cycle	7 of 1129	0.0000
GO:0051302	regulation of cell division	4 of 179	0.0001
GO:0051301	cell division	5 of 483	0.0001
GO:1901992	positive regulation of mitotic cell cycle phase transition	3 of 65	0.0003
GO:0007059	chromosome segregation	4 of 253	0.0003
GO:0007017	microtubule-based process	5 of 605	0.0003
GO:0000910	cytokinesis	3 of 71	0.0003
GO:0071897	DNA biosynthetic process	3 of 95	0.0005
GO:0006259	DNA metabolic process	5 of 773	0.0007
GO:0051782	negative regulation of cell division	2 of 16	0.0010
GO:0007051	spindle organization	3 of 123	0.0010
GO:0000819	sister chromatid segregation	3 of 123	0.0010
GO:0000226	microtubule cytoskeleton organization	4 of 393	0.0010
GO:0140014	mitotic nuclear division	3 of 136	0.0011
GO:0000075	cell cycle checkpoint	3 of 193	0.0027
GO:0006260	DNA replication	3 of 203	0.0028
GO:0007346	regulation of mitotic cell cycle	4 of 608	0.0036
GO:0019985	translesion synthesis	2 of 41	0.0042
GO:0010564	regulation of cell cycle process	4 of 684	0.0052
GO:0045840	positive regulation of mitotic nuclear division	2 of 54	0.0056
GO:0030071	regulation of mitotic metaphase/anaphase transition	2 of 49	0.0056
GO:0000281	mitotic cytokinesis	2 of 50	0.0056
GO:0033044	regulation of chromosome organization	3 of 313	0.0066
GO:0051225	spindle assembly	2 of 70	0.0076
GO:0007052	mitotic spindle organization	2 of 70	0.0076
GO:0051303	establishment of chromosome localization	2 of 72	0.0077

GO analyses were performed using STRING (https://string-db.org/). ^1^Benjamini-Hochberg-corrected p-value. Only the 30 most significant GO terms were reported.

Summary of the biological processes of GO terms of the 10 target genes that showed differential relative mRNA expression in lung CSCs comparing early stages (I-II) with advanced stages (IIIA) NSCLC.

## Discussion

Cancer stem cells are amongst the most debated causes of recurrence and resistance to anti-cancer treatments (26, 38–41). However, difficulties in identifying these cell populations in human tissue caused several limitations, especially for testing new drugs. Recently, Masciale et al. have suggested that there is a subpopulation of cells CD44+/EpCAM+ with a high correlation with ALDH^high^ cell populations (49) which were previously shown to be cancer stem cells in NSCLC patients (48). A marker like ALDH for cancer stem cells has also been identified and described by many scientists (50–52).

Additionally, the subject of NSCLC recurrence has been a source of significant interest because there is no effective treatment for lung cancer, and the five-year survival rate for lung cancer is 56% for cases detected when the disease is still localized (within the lungs) (53, 54). However, only 16% of lung cancer cases are diagnosed at an early stage. For distant tumors (spread to other organs), the five-year survival rate is only 5% (53).

The latest TNM guidelines (VIII Ed.) described the different stages for NSCLC based on tumor dimension (T), lymph node involvement (N), and other organ disseminations (M or metastasis) (9). At present, patients in the early stages are usually enrolled for surgical treatment with 5 years’ follow-up without medical therapies (9). The major problem is that early stages develop recurrence rather quickly, and more than 80% of recurrences occur within the first 2 years after resection (55). This aspect is one of the most crucial points in studies of recurrence that have been conducted in recent decades (56, 57). Since 2010, scientists have attempted to define a prognostic score to prevent recurrence in patients with early-stage adenocarcinoma of the lung. This is the most frequent histotype, occurring in 80%–85% of NSCLC (54–57).

In 2015, Bueno et al. from Brigham and Women Hospital (BWH) in collaboration with the Edinburgh College and Myriad Genetics Inc., isolated and validated a pool of genes mostly correlated with recurrence in a large population of patients harboring lung adenocarcinoma. They defined a prognostic score for early-stage adenocarcinoma (23). The possibility of applying this score for the early stages of lung cancer is highly promising, and it may be considered a milestone for new approaches for preventive treatments against recurrence in both short- and long-term periods. Supporting this scenario, Aramini et al. in 2016 confirmed that the CCP score was effective for predicting recurrence in patients who underwent surgery for early-stage adenocarcinoma of the lung (29).

This aspect has been recently discussed within a proposal of a new grading system for invasive pulmonary adenocarcinoma of the lung (58).

In light of the importance of CSC as a cause of recurrence and drug resistance, and supported by the knowledge of the pool of genes involved in recurrence (23), we designed our study to analyze 22 consecutive patients who underwent surgery for stage I, II, or IIIA NSCLC. From the surgical samples, we isolated the cancer stem cells (ALDH^high^ cells) and the cancer cells (ALDH^low^ cells) which had been recently studied by our group for adenocarcinoma and squamous cell carcinoma of the lung (48).

The main result of this research was that the pool of the previously validated cell cycle genes was highly overexpressed in ALDH^high^ cells when compared with ALDH^low^ cells in the early stages of NSCLC, whereas overexpression was lower in locally advanced stages of NSCLC. Regarding the higher expression of target genes in early stages (stage I/II), these genes are cell cycle–related and CSCs are characterized by self-renewal ability and uncontrolled proliferation, mainly responsible for the development of tumor initiation and progression (59). Together, these two conditions explain the higher level of gene expression in early stages, since a high proliferation rate is required, especially during the initial phase of progression, to sustain tumorigenesis and establish cellular heterogeneity within the primary tumor (60). According to our results, attention should be paid to the cell division pathway, particularly including PTTG1, TOP2A, CEP55, BIRC5, TK1, and ASPM genes that showed high overexpression in CSC (FC ≥ 8). Moreover, our results indicated that other genes associated with the cell cycle (CDK1, PRC1, ASF1B, CDKN3, DLGAP5, FOXM1, KIAA0101, CDCA3, KIF20A, NUSAP1, CENPM, PLK1, CDCA8, DTL, and CENPF) were overexpressed (FC ≥ 4) in lung cancer stem cells compared to in cancer cells. Although the cell cycle has been studied extensively at the molecular level since the end of the 20th century (61, 62), and because any perturbation in the expression of cell-cycle genes may lead to apoptosis or cancer (61), we did not focus on the mechanism of the cell-cycle genes. Rather, we aimed to determine if the dysregulation of those genes would also be present in cancer stem cells, representing the main cause of tumor relapse (63). Identifying overexpression of these genes in cancer stem cells could represent a significant opportunity for the treatment of lung cancer, both in the early and locally advanced stages. This may offer researchers the chance to block the specific gene or the regulated downstream processes that occurred in *in vitro* studies on prostate carcinoma, blocking PLK1 (64). Moreover, CDCA5 which was overexpressed in hepatocellular carcinoma (65), has been silenced with the consequent inhibition of the progression of tumors. Indeed, gene targeting is becoming a significant therapeutic modality with an increasing number of short interfering RNA drug approvals by the United States FDA. Nevertheless, there has been no attempt to silence genes in lung cancer stem cells as human HIF-2α was silenced in glioblastoma cancer stem cells (66).

In addition, even for locally advanced NSCLC, we identified a smaller pool of genes that were significantly overexpressed in ALDH^high^ cells. The study of these overexpressed genes could be helpful for the development of new target therapies, even in patients with locally advanced disease. Similarly, the resulted data on the overexpression in CSC of the cell cycle related genes may pave the way to new treatments ensuring a better prognosis in terms of tumor regression and survival for NSCLC patients.

Another interesting result from our study, although observed on a small number of patients, was that some genes showed differential overexpression in adenocarcinoma and squamous cell carcinoma of the lung. This aspect suggests a new approach for future treatments against cancer stem cells of each specific histotype.

### Limitations

The results of this study are limited in part by its observational design, making it impossible to rule out the possibility of selection bias or confounding bias. Moreover, the number of enrolled patients was mainly based on tissue and resource availability; therefore, it did not represent the optimal sample size. However, the analysis of paired NSCLC human tissues (ALDH^high^ and ALDH^low^) should have reduced the probability of a relevant confounding bias, and the selection bias should have been tempered by including all eligible cases in the considered period. Furthermore, this is the first study assessing the expression of CSC in human NSCLC tissue and its relationship with previously validated recurrence-related genes. It is also important to emphasize that the extraction, sorting, and mRNA analysis involved a multidisciplinary team and a significant amount of work. Nevertheless, the generalizability of our results is limited by the sample size; further studies in larger populations of early and locally advanced stage adenocarcinomas and squamous cell carcinomas are necessary to confirm our findings. Moreover, the direct relationship between the expression of these genes in CSC and the overall survival and relapse-free survival of NSCLC patients still needs to be assessed.

Another limitation relates to the use of ALDH as a marker for CSCs. Isolation of CSC has been challenging since the beginning, and several methods were used for their isolation. One of the most commonly used is flow cytometry (FCM) employing CSC-specific cell surface markers, like CD133 and CD44, and the ALDH1A1, which is the most used and described marker for lung cancer stem cells (67).

Finally, other limitations are that the pool of target genes analyzed was relatively small and that it was previously identified only for adenocarcinoma, while in this study we also assessed the target genes’ relative mRNA expression in squamous cell carcinoma. However, we observed similar results for these two histotypes, which may suggest a relevant role of the recurrence-related genes in patients harboring squamous cell carcinoma as well.

## Conclusion

Our study represents the first attempt towards identifying the genes associated with recurrence that are overexpressed in cancer stem cells in patients with early and locally advanced stages of NSCLC. Our results highlight the importance of overexpressed genes in cancer stem cells, which may be considered for new target therapies selected for NSCLC stages. In particular, basic experiments of CSC culture may be necessary for further studies to show the effectiveness of new treatment for targeting genes associated with the cell cycle (68). The future settings and development of new targeted drugs, using the most expressed genes in cancer stem cells for early or locally advanced stages, may represent a vital new approach to reduce recurrence, as well as to more effectively treat lung cancer patients.

## Data Availability Statement

The datasets presented in this study can be found in online repositories. The names of the repository/repositories and accession number(s) can be found below: NCBI Gene Expression Omnibus, https://www.ncbi.nlm.nih.gov/geo/ (GSE157427).

## Ethics Statement

The studies involving human participants were reviewed and approved by this Study, involving human subjects, human material, and human data, has been performed in accordance with the Declaration of Helsinki and has been approved by the Ethics committee at University Hospital of Modena, MODENA, Italy, on 17 March 2017, Prot. N. 914/C.E. Further information and documentation to support this is available to the Editor on request. The patients/participants provided their written informed consent to participate in this study.

## Author Contributions

The idea for the manuscript was conceived in September 2016 by BA and MD and was further developed by VM, GG, FB, RD’A, AM, AS, and AM were involved in histopathological diagnosis. BA, VM, and FB wrote the first draft of the manuscript. BA and UM have been involved in surgery and tissue collection. VM and GG performed laboratory experiments, whereas FB and RD’A performed the statistical analysis. BA, VM, FB, MD, RD’A, AM, and UM all reviewed the manuscript and were involved in its critical revision before submission. All authors contributed to the article and approved the submitted version.

## Funding

The Project has been supported in part by funds from the Division of Thoracic Surgery of the University Hospital of Modena and from the Laboratory of Cellular Therapy of the University of Modena and Reggio Emilia, from unrestricted grant from Myriad Inc. (US) and from the Italian Ministry of Education, University and Research: Departments of Excellence 2017.

## Conflict of Interest

The authors declare that the research was conducted in the absence of any commercial or financial relationships that could be construed as a potential conflict of interest.

## Abbreviations

ALDH, aldehyde dehydrogenase; ASF1B, anti-silencing function 1b histone chaperone; ASPM, assembly factor for spindle microtubules; BIRC5, baculoviral inhibitor of apoptosis repeat-containing 5; BUB1, budding uninhibited by benzimidazoles 1; CCP score, cell cycle proliferation; CDCA5, cell division cycle-associated gene 5; C18orf24, Chromosome 18 open reading frame 24; CDC20, cell-division cycle protein 20; CDCA3, cell division cycle-associated protein 3; CDCA8,cell division cycle associated 8; CDK1,cyclin-dependent kinase 1; CDKN3, cyclin-dependent kinase inhibitor 3; CENPF, centromere protein F; CENPM, centromere protein M; CEP55, centrosomal protein of 55; CSC, cancer stem cell; DLGAP5, disks large-associated protein 5; DTL, Diode–transistor logic; FACS, fluorescence-activated cell sorting; FC, fold change; FDR, false discovery rate; FOXM1, forkhead box protein M1; KIAA0101, PCNA-associated factor; KIF11, kinesin family member 11; KIF20A, kinesin-like protein 20A; LMM, linear mixed models; MCM10, protein MCM10 homolog; NSCLC, non-small cell lung cancer; NUSAP1, nucleolar and spindle associated protein 1; ORC6L, origin recognition complex subunit 6; PBK, lymphokine-activated killer T-cell-originated protein kinase; PLK1, Polo-like kinase 1; PRC1, protein regulator of cytokinesis 1; PTTG1, protein regulator of cytokinesis 1; RAD51, DNA repair and recombination protein RAD51-like; RAD54, DNA repair and recombination protein RAD54-like; RRM2, Ribonucleoside-diphosphate reductase subunit M2; RT-PCR, real time PCR; TK1, Thymidine kinase 1; TNM, tumor, node, metastasis; TOP2A, DNA topoisomerase 2-alpha.
