# Supplementary figures and images for: New Perspectives in Different Gene Expression Profiles for Early and Locally Advanced Non-Small Cell Lung Cancer Stem Cells
Source: Front Oncol. 2021 Mar 31;11:613198. doi: 10.3389/fonc.2021.613198 (PMC8047623; doi:10.3389/fonc.2021.613198)

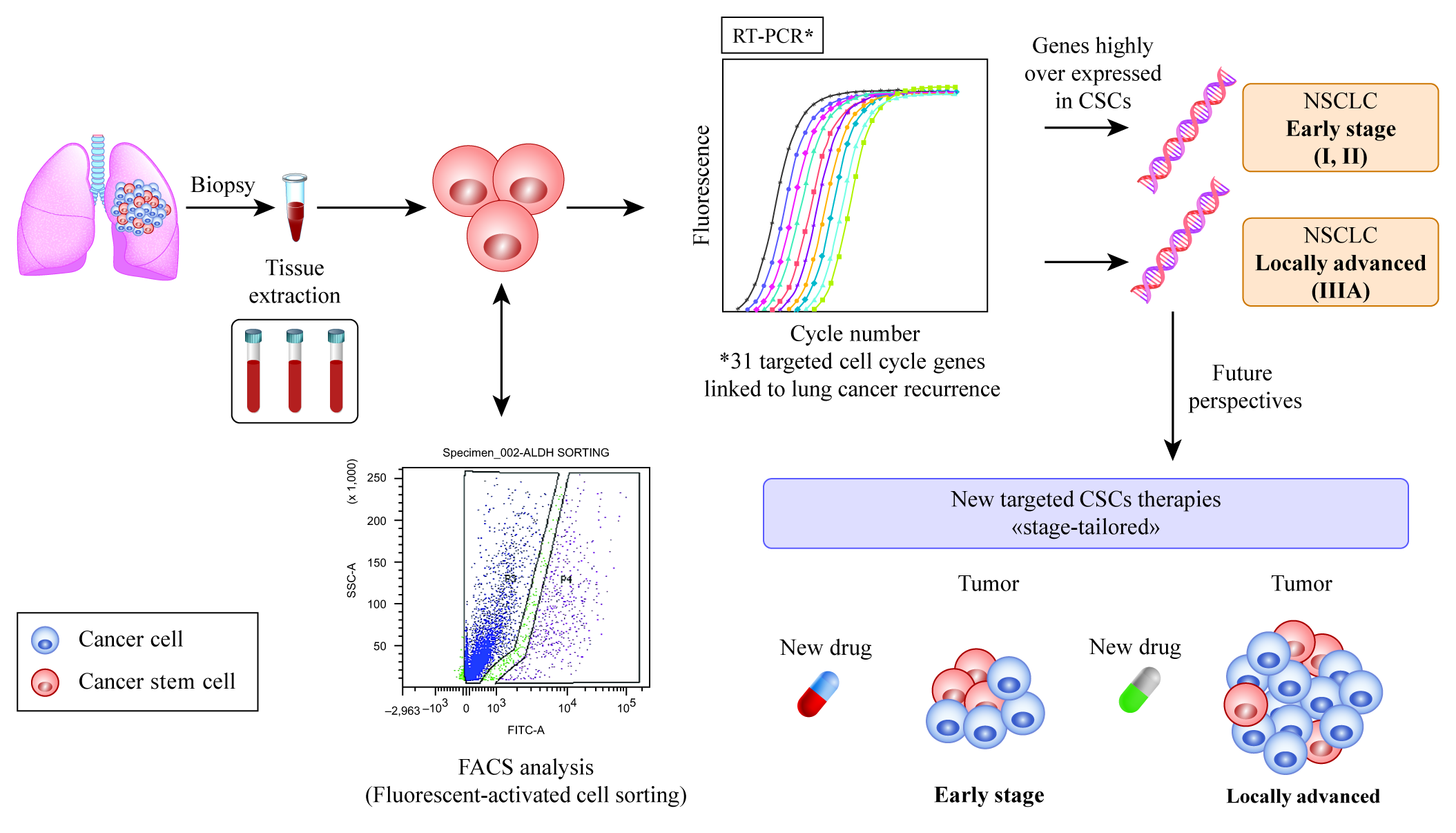

Supplement: Supplementary file 1 [file Image_1.tif]
